# Impact of timing of antiseizure medication withdrawal on seizure recurrence in glioma patients: a retrospective observational study

**DOI:** 10.1007/s11060-023-04450-z

**Published:** 2023-09-27

**Authors:** Pim B. van der Meer, Linda Dirven, Marta Fiocco, Maaike J. Vos, Melissa Kerkhof, Mathilde C.M. Kouwenhoven, Martin J. van den Bent, Martin J.B. Taphoorn, Johan A.F. Koekkoek

**Affiliations:** 1 Department of Neurology, Leiden University Medical Center, PO BOX 9600, 2300 RC Leiden, The Netherlands; 2 Department of Neurology, Haaglanden Medical Center, The Hague, The Netherlands; 3 Department of Biomedical Data Sciences, Medical Statistics, Leiden University Medical Center, Leiden, The Netherlands; 4 Mathematical Institute, Leiden University, Leiden, The Netherlands; 5 Department of Neurology, Amsterdam University Medical Center, Amsterdam, The Netherlands; 6 Department of Neurology, Erasmus Medical Center, Rotterdam, The Netherlands

**Keywords:** Glioma, Brain tumor, Antiepileptic drug, Seizure, Treatment failure

## Abstract

**Background:**

Withdrawal of antiseizure medication treatment (ASM) can be considered after completion of antitumour treatment in glioma patients who no longer suffer from seizures. We compared the risk for recurrent seizures after ASM withdrawal between patients with short-term, medium-term versus long-term seizure freedom after antitumour treatment.

**Methods:**

In this retrospective observational study, the primary outcome was time to recurrent seizure, from the starting date of no ASM treatment up to 36 months follow-up. Cox proportional hazards models were used to study the effect of risk factors on time to recurrent seizure. Stratification was done with information known at baseline. Short-term seizure freedom was defined as ≥ 3 months, but < 12 months; medium-term as 12–24 months; and long-term as ≥ 24 months seizure freedom from the date of last antitumour treatment.

**Results:**

This study comprised of 109 patients; 31% (34/109) were in the short-term, 29% (32/109) in the medium-term, and 39% (43/109) in the long-term group. A recurrent seizure was experienced by 47% (16/34) of the patients in the short-term, 31% (10/32) in the medium-term, and 44% (19/43) in the long-term group. Seizure recurrence risk was similar between patients in the short-term group as compared to the medium-term (cause-specific adjusted hazard ratio [aHR] = 0.65 [95%CI = 0.29–1.46]) and long-term group (cause-specific aHR = 1.04 [95%CI = 0.52–2.09]).

**Conclusions:**

Seizure recurrence risk is relatively similar between patients with short-term, medium-term, and long-term seizure freedom after completion of antitumour treatment.

**Supplementary Information:**

The online version contains supplementary material available at 10.1007/s11060-023-04450-z.

## Introduction

Epileptic seizures are a common symptom in glioma patients, for which antiseizure medication (ASM) treatment is standard-of-care [1]. ASM treatment is often accompanied by adverse effects (up to 90% when intensively monitored), [2] leading to discontinuation of the ASM in ~ 15–20% of glioma patients [3, 4]. At some point during the course of the disease, a risk-benefit evaluation can be made by the physician with regard to the continuation of ASM treatment, leading to a shared decision together with the patient that withdrawal of the ASM might be a viable option. In other cases patients might be so burdened by the adverse effects of the ASM that they make their own decision to discontinue ASM treatment. A prospective study in glioma patients showed that 26% (12/46) of carefully selected grade 2 or grade 3 glioma patients by physicians (≥ 12 months seizure free from the date of last antitumour treatment or ≥ 24 months seizure free if a seizure occurred after the date of last antitumour treatment) had a recurrent seizure in 1.5 years after ASM withdrawal compared to 8% (2/25) of patients continuing ASM treatment [5]. Similar recurrent seizure rates were reported in retrospective ASM withdrawal studies in which no specific criteria were used to initiate ASM withdrawal in brain tumour patients. In adult brain tumour patients with epilepsy (median follow-up 3.1 years) 19% (3/16) had a recurrent seizure and in child brain tumour patients with epilepsy (median follow-up 2.3 years) 27% (17/62) had a recurrent seizure after ASM withdrawal [6, 7]. Nevertheless, these estimates should be interpreted with caution as in these latter studies several methodological issues were not taken into account (e.g., the competing risk of death, modelling time to event with appropriate analysis, inclusion of predictors such as antitumour treatment and tumour characteristics).

Currently, it is unknown whether recurrent seizure rates depend on the period of seizure freedom. In adult non-brain tumour-related epilepsy (non-BTRE), ASM withdrawal is in most studies considered in patients at least two year seizure free, but compelling evidence is lacking for optimal timing of ASM withdrawal [8, 9]. Information on the impact of timing of ASM withdrawal on the risk of seizure recurrence may help both patients and physicians to make well-informed decisions to either continue or withdraw ASM treatment. This study aimed to evaluate seizure recurrence rates after ASM withdrawal in glioma patients with short-term, medium-term and long-term seizure freedom.

## Methods

### Study population and procedures

In a previously described cohort (n = 1435), we included consecutive adult (≥ 18 years) patients, with a histologically confirmed diffuse glioma according to the 2016 World Health Organization (WHO) classification of tumours of the central nervous system (diagnoses were updated according to the 2021 WHO classification for the current study), [10] who had either a biopsy or surgical (re)resection in one of three large referral neuro-oncology outpatient clinics in the Netherlands, between January 1st, 2004, and January 1st, 2018, and who had received first-line ASM treatment with monotherapy levetiracetam or valproic acid after the occurrence of an epileptic seizure. A more elaborate description of the methodology can be found elsewhere [4]. For the current analysis, all patients who at some point during their disease trajectory after the initiation of their first-line ASM treatment had no ASM treatment (i.e., because of withdrawal due to remission of seizures, poor adherence, intolerable adverse effects, or other reasons), had completed antitumour treatment (i.e., subtotal or gross total surgical resection, radiotherapy, and/or chemotherapy), and were seizure free for ≥ 3 months, were included. Meaning, patients were allowed to have used ASM polytherapy treatment at some point during their disease trajectory as long as they had no ASM treatment at some point. Baseline information (i.e., starting date of no ASM treatment) was collected through examining medical charts concerning sociodemographic characteristics, tumour and treatment characteristics, presence of radiological progressive disease (≤ 3 months before or after time of recurrent seizure) based on the Response Assessment in Neuro-Oncology (RANO) criteria, [11] and information on seizure characteristics. The medical ethics committee of each institution approved the protocol and consent of patients was obtained according to institutional policies.

### Outcomes

Time to recurrent seizure, from the starting date of no ASM treatment, was the primary outcome in this study. Secondary outcome was time to restart ASM treatment due to seizures, from the starting date of ASM discontinuation, to evaluate to what extent patients are willing to restart ASM treatment after the occurrence of a recurrent seizure. Time to recurrent seizure, from the starting date of ASM discontinuation, was also estimated and compared between the different tumour grades (grade 2 or grade 3 versus grade 4). Maximum duration of follow-up was 36 months.

### Statistics

Stratification was done with information known at baseline. Short-term seizure freedom rates (i.e., ≥ 3 months, but < 12 months seizure freedom from the date of last antitumour treatment) were compared with medium-term seizure freedom rates (i.e., ≥ 12 months, but < 24 months seizure freedom from the date of last antitumour treatment) and long-term seizure freedom rates (i.e., ≥ 24 months seizure freedom from the date of last antitumour treatment). The following groups were made partly based on the inclusion criteria from a recent prospective ASM withdrawal study by our research group [5]. Given the limited survival time of glioma patients, patients may die before reaching the maximal follow-up of 36 months or the outcome of interest (i.e., seizure recurrence or restart of ASM treatment) [12]. To estimate the cumulative incidence function for time to recurrent seizure and time to restart ASM treatment, two competing risks models were estimated: (1) recurrent seizure and death as competing event; and (2) restart ASM treatment and death as competing event [13]. The Gray test was used to assess differences between the cumulative incidences [14].

Cause-specific Cox proportional hazards regression models were used to study the effects of potential prognostic factors on time to recurrent seizure and time to restart ASM treatment. A directed acyclic graph (DAG) representation was used to identify potential confounders based on pre-existing knowledge. A confounder should be associated with both the predictor (i.e., the different seizure freedom groups) and the outcome (i.e., time to recurrent seizure and time to restart ASM treatment), but not lay in the causal pathway [15]. The following baseline characteristics were included as potential confounders in the Cox model: tumour grade, isocitrate dehydrogenase (IDH)-mutation status, previous antitumour treatment (surgical resection, radiotherapy and/or chemotherapy therapy), and tumour location. Based on results of simulation studies, at least five events per selected variable for the Cox model were required [16]. Violation of the proportional hazards assumption was investigated by looking at the Schoenfeld residuals and performing proportional hazards tests [17].

The chi-square test was used to analyse presence of radiological tumour progression when a recurrent seizure occurred between the short-term, medium-term, and long-term seizure freedom group. The Kaplan-Meier estimator was used to estimate progression-free survival (time since radiological diagnosis) for the short-term, medium-term, and long-term seizure freedom group and the Log-rank test to compare survival curves between the three groups. In addition, cumulative incidences of time to recurrent seizure, presence of radiological tumour progression when a recurrent seizure occurred, and progression-free survival were estimated and compared between the different tumour grades. Based on the DAG representation, potential confounders when evaluating the effect of duration of seizure freedom on seizure recurrence were no potential confounders when evaluating the effect of tumour grade on seizure recurrence, because these baseline variables lay in the causal pathway (e.g., tumour grade is not affected by radiotherapy and/or chemotherapy, but tumour grade might affect whether radiotherapy and/or chemotherapy is given and has subsequently an effect on seizure recurrence). Therefore, the unadjusted hazard ratio (uHR) was used opposed to the adjusted hazard ratio (aHR) when comparing seizure recurrence between grade 2 or grade 3 and grade 4 glioma. All competing risks analyses were done in R software with the cmprsk library, other analyses were done in SPSS software version 25.0 [18, 19]. Statistical significance was set at a p-value of < 0.05.

## Results

### Patient characteristics

**Fig. 1:**
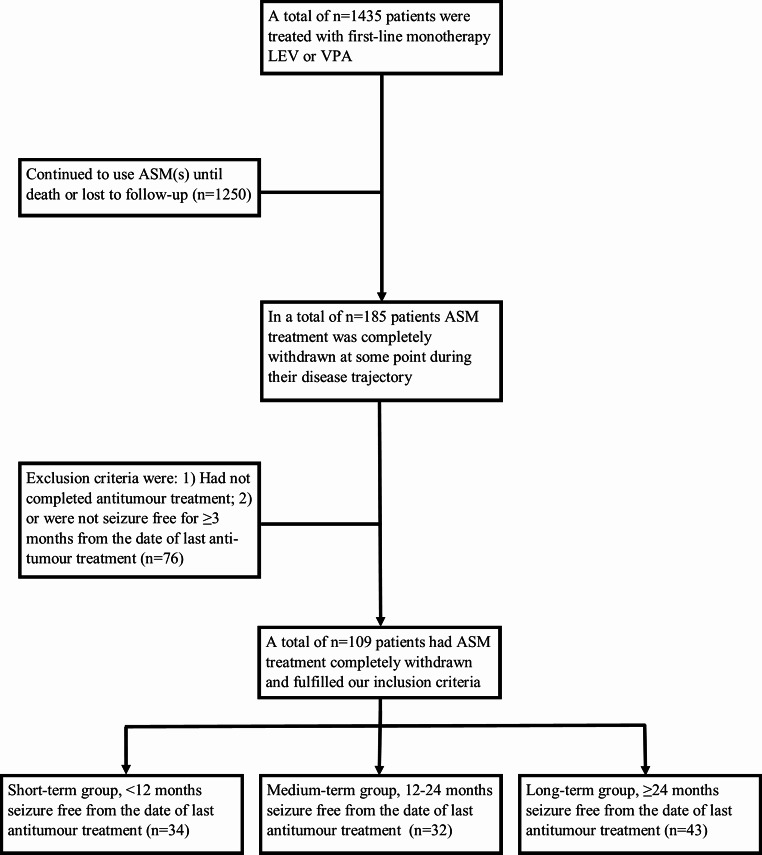
Flowchart of in- and excluded patients

In n = 185 patients ASM treatment was completely discontinued during their disease trajectory, but only n = 109 (59%) patients fulfilled our inclusion criteria (Fig. 1). A total of 31% (34/109) of patients were < 12 months seizure free from the date of last antitumour treatment (the short-term group), 29% (32/109) 12–24 months seizure free (the medium-term group), and 39% (43/109) ≥ 24 months seizure free (the long-term group). Baseline demographic characteristics of the three groups are reported in Table 1. Most patients were > 40 years, were male, had a grade 2 glioma, underwent surgical resection, received radiotherapy, had focal to bilateral tonic-clonic seizures, were only treated with ASM monotherapy, and never had a status epilepticus. Only sex differed significantly between the three groups, with less males in the long-term (44% [19/43]) compared to the medium-term seizure freedom group (78% [25/32], p = 0.003), but not compared to the short-term seizure freedom group (56% [19/34], p = 0.308) or between the short-term and medium-term seizure freedom group (p = 0.055).

**Table 1:** Baseline demographic characteristics of the patients after antiseizure medication withdrawal

	Antiseizure medication (ASM) treatment withdrawal			
Characteristics	Short-term^1^	Medium-term^2^	Long-term^3^	P-value
Patients included, no. (%)	34 (100)	32 (100)	43 (100)	
Age, no. (%)				0.211
≤40 years	13 (38)	13 (41)	10 (23)	
>40 years	21 (62)	19 (59)	33 (77)	
Sex, no. (%)				0.013
Male	19 (56)	25 (78)	19 (44)	
Female	15 (44)	7 (22)	24 (56)	
Tumour grade and pathology, no. (%)				0.102
Grade 2	18 (53)	22 (69)	26 (60)	
Diffuse astrocytoma, NOS	1 (3)	4 (13)	9 (21)	
Diffuse astrocytoma, IDH-mutant	7 (21)	8 (25)	4 (9)	
Oligodendroglioma, NOS	2 (6)	2 (6)	1 (2)	
Oligodendroglioma, IDH-mutant 1p/19q codeletion	8 (24)	8 (25)	11 (26)	
Pleiomorphic xanthroastrocytoma	0 (0)	0 (0)	1 (2)	
Grade 3	7 (21)	5 (16)	14 (33)	
Diffuse astrocytoma, NOS	3 (9)	1 (3)	2 (5)	
Diffuse astrocytoma, IDH-mutant	2 (6)	0 (0)	3 (7)	
Oligodendroglioma, NOS	0 (0)	1 (3)	0 (0)	
Oligodendroglioma, IDH-mutant 1p/19q codeletion	2 (6)	3 (9)	8 (19)	
Oligoastrocytoma, NOS	0 (0)	0 (0)	1 (2)	
Grade 4	9 (26)	5 (16)	3 (7)	
Glioblastoma, NOS	6 (18)	3 (9)	2 (5)	
Glioblastoma, IDH-wildtype	2 (6)	0 (0)	0 (0)	
Diffuse astrocytoma, IDH-mutant	1 (3)	2 (6)	1 (2)	
Surgical resection prior to ASM withdrawal, no. (%)				0.202
Yes	33 (97)	31 (97)	38 (88)	
No (including biopsy)	1 (3)	1 (3)	5 (12)	
Radiotherapy prior to ASM withdrawal, no. (%)				0.444
Yes	26 (76)	20 (63)	31 (72)	
No	8 (24)	12 (38)	12 (28)	
Systemic therapy prior to ASM withdrawal, no. (%)				0.100
Yes	19 (56)	10 (31)	16 (37)	
Temozolomide	12 (35)	7 (22)	14 (33)	
Temozolomide rechallenge	1 (3)	0 (0)	1 (2)	
PCV^4^	7 (21)	4 (13)	4 (9)	
Lomustine	2 (6)	0 (0)	0 (0)	
No	15 (44)	22 (69)	27 (63)	
Tumour involvement in the temporal lobe				0.554
Yes	15 (44)	11 (34)	20 (47)	
No	19 (56)	21 (66)	23 (53)	
Seizure type, no. (%)				0.596
Focal	9 (26)	9 (28)	13 (30)	
Focal to bilateral tonic-clonic^5^	24 (71)	21 (66)	30 (70)	
Unknown	1 (3)	2 (6)	0 (0)	
Previously treated ASM regimen, no. (%)				0.098
Monotherapy	33 (97)	27 (84)	41 (95)	
Polytherapy	1 (3)	5 (16)	2 (5)	
Status epilepticus prior to ASM withdrawal, no. (%)				0.300
Yes	5 (15)	4 (13)	2 (5)	
No	29 (85)	28 (88)	41 (95)	

^1^Patients were ≥ 3 months, but < 12 months seizure free from the date of last antitumour treatment; ^2^patients were 12–24 months seizure free from the date of last antitumour treatment; ^3^Patients were ≥ 24 months seizure free from the date of last antitumour treatment; ^4^PCV=Procarbazine, Lomustine, and Vincristine; ^5^Patients had either solely focal to bilateral tonic-clonic seizures or both focal and focal to bilateral tonic-clonic seizures; No.=Number of patients

Reasons for ASM discontinuation differed between patients in the short-term, medium-term, and long-term seizure freedom group (eTable 1). In most patients ASM withdrawal was initiated when patients were using first-line monotherapy levetiracetam or valproic acid (62% [21/34] in the short-term, 69% [22/32] in the medium-term, and 49% [21/43] in the long-term seizure freedom group). Median time of seizure freedom since antitumour treatment before ASM withdrawal was 6.4 months (interquartile range [IQR] = 4.7–9.6 months) in the short-term, 16.5 months (IQR = 13.6–19.4 months) in the medium-term, and 40.6 months (IQR = 32.6–52.0 months) in the long-term seizure freedom group. Median number of months from first epileptic seizure ever to ASM withdrawal was 15.9 months (IQR = 8.3–24.4 months) in the short-term, 29.7 months (IQR = 24.2–40.5 months) in the medium-term, and 62.1 months (IQR = 45.0-101.9 months) in the long-term seizure freedom group. Median progression-free survival time was 38.8 months (95%CI = 13.4–64.2 months) in the short term, 80.7 months (95%CI = 45.4–116.0 months) in the medium-term, and 121.1 months (95%CI = 102.2-140.1 months, p = 0.015) in the long-term seizure freedom group. Of note, median progression-free survival time was 93.5 months (IQR = 59.8-127.3 months) in grade 2, 116.1 months (IQR = 83.7-148.5 months) in grade 3, and 34.6 months (IQR = 13.9–55.3 months, p = 0.025) in grade 4 glioma. During the follow-up period after ASM withdrawal was initiated, 12% (4/34) received antitumour treatment in the short-term, 6% (2/32) in the medium-term, and 9% (4/43) in the long-term seizure freedom group.

### Time to recurrent seizure

**Fig. 2:**
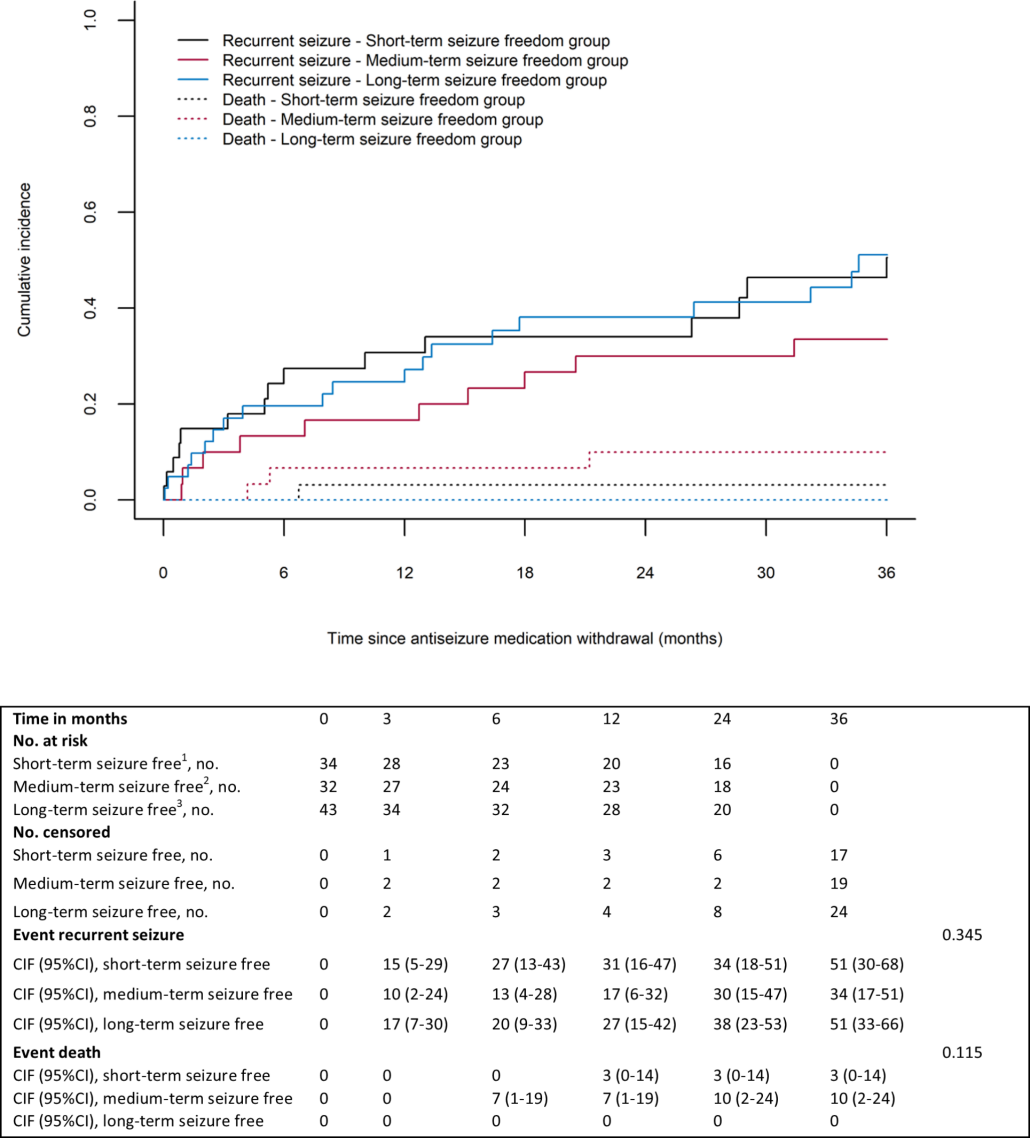
Cumulative incidences of time to recurrent seizure, from moment of antiseizure medication withdrawal: short-term versus medium-term versus long-term seizure freedom ^1^Patients were ≥ 3 months, but < 12 months seizure free from the date of last antitumour treatment; ^2^Patients were 12–24 months seizure free from the date of last antitumour treatment; ^3^Patients were ≥ 24 months seizure free from the date of last antitumour treatment; CI = Confidence interval; CIF = Cumulative incidence function; No.=Number of patients

During 36 months of follow-up 47% (16/34) of the patients in the short-term, 31% (10/32) in the medium-term, and 44% (19/43) in the long-term seizure freedom group experienced a recurrent seizure. The cumulative incidence of a recurrent seizure at 12 months for the short-term, medium-term, and long-term seizure freedom group was equal to 31% (95%CI = 16–47%), 17% (95%CI = 6–32%), and 27% (95%CI = 15–42%, p = 0.345), respectively (Fig. 2). The risk for a recurrent seizure was similar between patients in the short-term and medium-term seizure freedom group (cause-specific aHR = 0.65 [95%CI = 0.29–1.46]) and between the short-term and long-term seizure freedom group (cause-specific aHR = 1.04 [95%CI = 0.52–2.09] [Table 2]). The percentage of patients with radiological tumour progression at time of the recurrent seizure was not significantly different between the short-term, medium-term, and long-term seizure freedom group (19% [3/16] versus 30% [3/10] versus 16% [3/19], p = 0.653).

**Table 2:** Unadjusted and adjusted hazard ratio’s of time to recurrent seizure

		Recurrent seizure			
Parameter		uHR (95% CI)	p-value	aHR (95% CI)	p-value
Antiseizure medication (ASM) withdrawal	Short-term seizure free^1^ (ref.)				
	Medium-term seizure free^2^	0.61 (0.27–1.36)	0.228	0.65 (0.29–1.46)	0.297
	Long-term seizure free^3^	0.95 (0.48–1.86)	0.869	1.04 (0.52–2.09)	0.914
Isocitrate dehydrogenase (IDH)-mutation	No (ref.)				
	Yes	0.85 (0.46–1.57)	0.596	1.27 (0.62–2.57)	0.514
Tumour grade	Grade 2 and grade 3 (ref.)				
	Grade 4	3.01 (1.47–6.17)	0.003*	2.32 (1.00-5.37)	0.050
Surgical resection prior to ASM withdrawal	No (including biopsy, ref.)				
	Yes	1.11 (0.34–3.59)	0.862	1.82 (0.53–6.28)	0.346
Radiotherapy and/or chemotherapy prior to ASM withdrawal	No (ref.)				
	Yes	1.49 (0.73–3.01)	0.271	1.32 (0.61–2.85)	0.475
Tumour involvement in the temporal lobe	No (ref.)				
	Yes	1.35 (0.75–2.45)	0.319	1.43 (0.75–2.70)	0.275

^1^Patients were ≥ 3 months, but < 12 months seizure free from the date of last antitumour treatment; ^2^patients were 12–24 months seizure free from the date of last antitumour treatment; ^3^Patients were ≥ 24 months seizure free from the date of last antitumour treatment; *P-value < 0.05; aHR = Adjusted hazard ratio; CI = Confidence interval; uHR = Unadjusted hazard ratio

A recurrent seizure was experienced by 39% (36/92) of grade 2 or grade 3 and 53% (9/17) of grade 4 glioma during 36 months of follow-up. The cumulative incidence of a recurrent seizure at 12 months for grade 2 or grade 3 and grade 4 glioma was equal to 22% (95%CI = 14–31%) and 42% (95%CI = 18–64%, p = 0.058), respectively. The risk for a recurrent seizure differed significantly between grade 2 or grade 3 versus grade 4 glioma (cause-specific uHR = 3.01 [95%CI = 1.47–6.17]). Radiological tumour progression at time of the recurrent seizure was significantly more often present in grade 4 glioma (56% [5/9]) compared to grade 2 or grade glioma (11% [4/36], p = 0.003).

### Time to restart ASM treatment

**Fig. 3:**
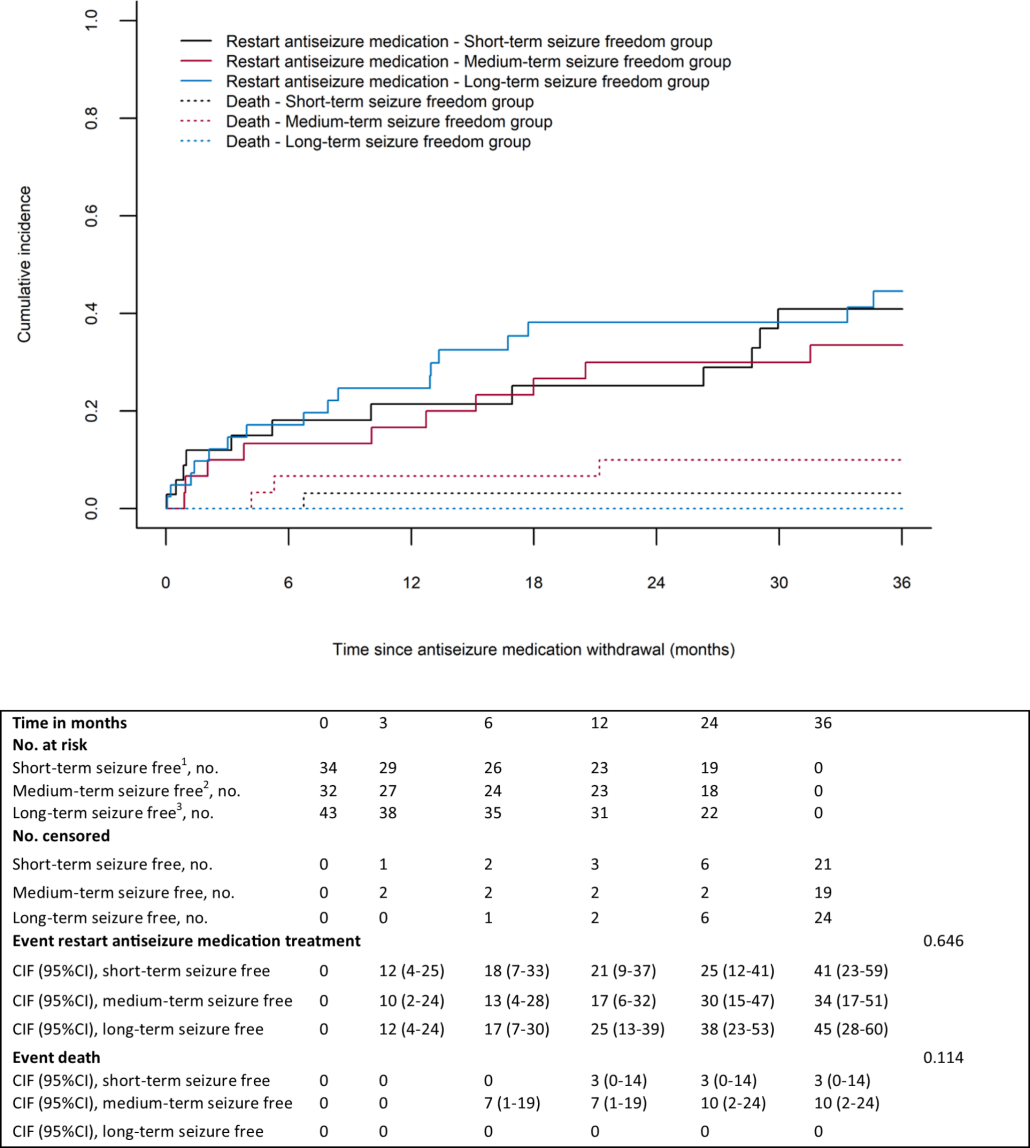
Cumulative incidences of time to restart antiseizure medication treatment, from moment of antiseizure medication withdrawal: short-term versus medium-term versus long-term seizure freedom ^1^Patients were ≥ 3 months, but < 12 months seizure free from the date of last antitumour treatment; ^2^patients were 12–24 months seizure free from the date of last antitumour treatment; ^3^Patients were ≥ 24 months seizure free from the date of last antitumour treatment; CI = Confidence interval; CIF = Cumulative incidence function; No.=Number of patients

The vast majority of the patients in both the short-term, medium-term, and long-term seizure freedom group directly (i.e., in < 3 months) restarted ASM treatment after having had a recurrent seizure (73% [11/15] versus 90% [9/10] versus 79% [15/19]). Reasons for not immediately restarting ASM treatment were mainly due to patients’ perceived barriers. The cumulative incidence of restarting ASM treatment at 12 months for the short-term, medium-term, and long-term seizure freedom group was equal to 21% (95%CI = 9–37%), 17% (95%CI = 6–32%), and 25% (95%CI = 13–39%), respectively (Fig. 3). No difference was found between the short-term and medium-term seizure freedom group (cause-specific aHR = 0.88 [95%CI = 0.37–2.06]) and between the short-term and long-term seizure freedom group (cause-specific aHR = 1.36 [95%CI = 0.64–2.91]) with regard to restarting ASM treatment (Table 3). ASM treatment was restarted in 86% (31/36) in grade 2 or grade 3 and 89% (8/9) in grade 4 glioma patients.

**Table 3:** Unadjusted and adjusted hazard ratio’s of time to restart antiseizure medication treatment

		Restart antiseizure medication (ASM) treatment			
Parameter		uHR (95% CI)	p-value	aHR (95% CI)	p-value
Antiseizure medication (ASM) withdrawal	Short-term seizure free^1^ (ref.)				
	Medium-term seizure free^2^	0.84 (0.36–1.94)	0.683	0.88 (0.37–2.06)	0.763
	Long-term seizure free^3^	1.14 (0.55–2.39)	0.726	1.36 (0.64–2.91)	0.428
Isocitrate dehydrogenase (IDH)-mutation	No (ref.)				
	Yes	0.97 (0.51–1.85)	0.928	1.47 (0.69–3.11)	0.321
Tumour grade	Grade 2 and grade 3 (ref.)				
	Grade 4	2.28 (1.04–4.99)	0.040*	2.50 (1.02–6.15)	0.046
Surgical resection prior to ASM withdrawal	No (including biopsy, ref.)				
	Yes	1.45 (0.35-6.00)	0.612	2.25 (0.51–9.98)	0.284
Radiotherapy and/or chemotherapy prior to ASM withdrawal	No (ref.)				
	Yes	1.22 (0.59–2.50)	0.591	1.11 (0.50–2.43)	0.801
Tumour involvement in the temporal lobe	No (ref.)				
	Yes	1.23 (0.66–2.33)	0.516	1.40 (0.71–2.76)	0.333

^1^Patients were ≥ 3 months, but < 12 months seizure free from the date of last antitumour treatment; ^2^patients were 12–24 months seizure free from the date of last antitumour treatment; ^3^Patients were ≥ 24 months seizure free from the date of last antitumour treatment; *P-value < 0.05; aHR = Adjusted hazard ratio; CI = Confidence interval; uHR = Unadjusted hazard ratio

## Discussion

Results of this study indicate that if ASM treatment is withdrawn in glioma patients, the risk of recurrent seizure may not be dependent on the duration of seizure freedom after completion of antitumour treatment. Most patients restart ASM treatment after having had a recurrent seizure to improve seizure control. Although only a limited number of grade 4 glioma patients were included in this analysis, they had the highest risk of having a recurrent seizure, most likely related to the occurrence of disease progression. The treating physician of glioma patients with epilepsy needs to make a risk-benefit evaluation with regard to ASM treatment. ASM withdrawal in grade 4 glioma patients should be discouraged given their high risk of a recurrent seizure in combination with their poor prognosis, meaning there is only a limited period they are likely to benefit from having no ASM treatment (i.e., no potential adverse effects). On the other hand, grade 2 and grade 3 glioma patients might benefit after ASM withdrawal for years, or even a decade, from not having adverse effects due to ASM treatment, which may increase their overall health-related quality of life [2, 20]. However, the number of grade 4 glioma patients in our study was low, so results should be interpreted with caution. Duration of seizure freedom after antitumour treatment does not seem to be an important prognostic risk factor for a recurrent seizure. This means that if the physician decides together with the patient withdrawal of the ASM might be a viable option after antitumour treatment, there seems to be no need to wait at least 1–2 years, but ASM withdrawal can be considered after 3 months of seizure freedom.

Reasons for ASM withdrawal in this study differed considerably between patients with short-term compared to medium-term or long-term seizure freedom. In the first group the majority of patients withdrew their ASM because of intolerable adverse effects or due to poor adherence, while in the latter two groups ASM withdrawal was initiated because of supposed seizure remission by the treating physician. However, this difference in reason for ASM withdrawal did not result in a difference in risk of recurrent seizure between the three seizure freedom groups. Potentially, the risk of a recurrent seizure would be even lower in the short-term seizure freedom group if more patients were carefully selected by the treating physician opposed to ASM withdrawal because of intolerable adverse effects or a lack of ASM treatment adherence.

Whether ASM treatment should be withdrawn after completion of antitumour treatment, depends on numerous alternative factors and should be based on a process of shared decision making between the patient and physician. In previous prospective and retrospective observational ASM withdrawal studies in adult glioma patients, grade 4 glioma patients were purposely excluded from participation [5, 6]. Therefore, risk of seizure recurrence after ASM withdrawal in grade 4 glioma was unknown, but based on the results from our study withdrawal of ASM treatment in grade 4 glioma should generally be discouraged. The cumulative incidence of seizure recurrence in the short-term, medium-term, and long-term seizure freedom group was comparable to the cumulative recurrence rate in a meta-analysis in only ASM treated non-BTRE patients after withdrawal at 12 months (17–31% versus 22%), 24 months (30–38% versus 28%), and 36 or 48 months of follow-up (34–51% versus 34%). However, in surgically treated non-BTRE patients cumulative incidences of seizure recurrence were generally lower (i.e., 14% seizure recurrence at 12 months, 21% at 24 months, and 24% at 36 or 48 months of follow-up) [21]. Physicians treating glioma patients should be aware of the greater risk of seizure recurrence after ASM withdrawal, even if only patients with an assumed low risk of seizure recurrence (such as patients with long-term seizure freedom and stable disease) are selected. For this reason, ASM withdrawal in glioma patients seems somewhat less attractive compared to non-BTRE patients and a thorough consideration of the risks and benefits of ASM withdrawal together with the patient is of even greater importance. Duration of seizure freedom after antitumour treatment does not seem to be an important prognostic risk factor for a recurrent seizure. Of note, in most patients the recurrent seizure was not related to tumour progression.

### Limitations

Due to the retrospective observational design of our study, the decision to withdraw ASM treatment was not controlled and patients withdrew from their ASM treatment for different reasons (e.g., poor adherence, intolerable adverse effects). We adjusted in our Cox model for important known confounders, but potential residual confounding of unmeasured confounders might still be present. Patients might not be using ASM treatment, but they might have used (non-approved) therapies instead, such as ketogenic diet or cannabidiol/tetrahydrocannabinol oil, which might have a beneficial effect on seizure control as well. Although we did include certain epilepsy characteristics (e.g., seizure type), which did not differ significantly between the groups, other potentially important epilepsy data (e.g., electroencephalogram data) was not available and this might have influenced results. In addition, our sample size was limited and therefore confidence intervals are wide. The lack of finding a statistically significant difference between the three seizure freedom groups might be due to a lack of power given the difference in a recurrent seizure at 12 months between the medium-term and the short-term or long-term seizure freedom group is ≥ 10%. Still, we do think we have a reasonable sample size given the relative rarity of the disease and the small percentage of patients withdrawing ASM treatment.

## Conclusion

To conclude, risk of recurrent seizure seems relatively similar between patients with short-term, medium-term, and long-term seizure freedom after completion of antitumour treatment. Therefore, waiting for a patient to be at least 1 or 2 years seizure free if ASM withdrawal is considered does not seem to be indicated in glioma patients with epilepsy. Most patients with a recurrent seizure did not have tumour progression at time of seizure recurrence. ASM withdrawal might be an option in certain patients after antitumour treatment, e.g., patients suffering from intolerable adverse effects. Our results suggest that ASM withdrawal could particularly be considered in a patient with a grade 2 or grade 3 glioma. In contrast, ASM treatment withdrawal in grade 4 glioma patients should be discouraged given their high risk of recurrent seizure and expected poor survival time. However, seizure recurrence during 36 months was relatively high for all groups (range 31–47%) and therefore treatment modification rather than ASM discontinuation is recommended.

### Electronic supplementary material

Below is the link to the electronic supplementary material.

Supplementary Material 1

## Data Availability

Data are available from the corresponding author upon reasonable request.
