# Supplementary material for: Impact of timing of antiseizure medication withdrawal on seizure recurrence in glioma patients: a retrospective observational study
Source: J Neurooncol. 2023 Sep 27;164(3):545–55. doi: 10.1007/s11060-023-04450-z (PMC10589365; doi:10.1007/s11060-023-04450-z)
Supplement: Supplementary file 1 — Supplementary Material 1 [file 11060_2023_4450_MOESM1_ESM.docx]

**Supplementary Material**

**Impact of timing of antiseizure medication withdrawal on seizure recurrence in glioma patients: a retrospective observational study**

Pim B. van der Meer, Linda Dirven, Marta Fiocco, Maaike J. Vos, Mathilde C.M. Kouwenhoven, Martin J. van den Bent, Martin J.B. Taphoorn, Johan A.F. Koekkoek

**Content:**

**eTable 1.** Demographic characteristics of the patients at baseline for the different reasons of antiseizure medication withdrawal

**Supplementary** **Table 1.** Demographic characteristics of the patients at baseline for the different reasons of antiseizure medication withdrawal

|  | **Reasons for ASM treatment withdrawal** | | | | |
| --- | --- | --- | --- | --- | --- |
| **Characteristics** | **Adverse effects** | **Poor adherence** | **Withdrawal because of supposed seizure remission by physician** | **Other reasons** | **P-value** |
| Patients included, no. (%) | 29 (100) | 15 (100) | 58 (100) | 7 (100) |  |
| ASM treatment withdrawal group, no. (%) |  |  |  |  | <0.001 |
| Short-term | 15 (52) | 10 (67) | 8 (14) | 1 (14) |  |
| Medium-term | 5 (17) | 2 (13) | 24 (41) | 1 (14) |  |
| Long-term | 9 (31) | 3 (20) | 26 (45) | 5 (71) |  |
| Age, no. (%) | | | | | 0.754 |
| ≤40 years | 10 (34) | 5 (33) | 20 (34) | 1 (14) |  |
| >40 years | 19 (66) | 10 (67) | 38 (66) | 6 (86) |  |
| Sex, no. (%) | | | | | 0.765 |
| Male | 17 (59) | 10 (67) | 33 (57) | 3 (43) |  |
| Female | 12 (41) | 5 (33) | 25 (43) | 4 (57) |  |
| Tumour grade and pathology, no. (%) | | | | | 0.797 |
| Grade 2 | 18 (62) | 8 (53) | 36 (62) | 4 (57) |  |
| Grade 3 | 7 (24) | 3 (20) | 15 (26) | 1 (14) |  |
| Grade 4 | 4 (14) | 4 (27) | 7 (12) | 2 (29) |  |
| Surgical resection prior to ASM withdrawal, no. (%) | | | | | 0.331 |
| Yes | 28 (97) | 15 (100) | 52 (90) | 7 (100) |  |
| No (including biopsy) | 1 (3) | 0 (0) | 6 (10) | 0 (0) |  |
| Radiotherapy prior to ASM withdrawal, no. (%) | | | | | 0.382 |
| Yes | 21 (72) | 8 (53) | 42 (72) | 6 (86) |  |
| No | 8 (28) | 7 (47) | 16 (28) | 1 (14) |  |
| Systemic therapy prior to ASM withdrawal, no. (%) | | | | | 0.458 |
| Yes | 15 (52) | 7 (47) | 20 (34) | 3 (43) |  |
| No | 14 (48) | 8 (53) | 38 (66) | 4 (57) |  |
| Tumour involvement in the temporal lobe | | | | | 0.327 |
| Yes | 12 (41) | 9 (60) | 21 (36) | 4 (57) |  |
| No | 17 (59) | 6 (40) | 37 (64) | 3 (43) |  |
| Seizure type, no. (%) | | | | | 0.796 |
| Focal | 8 (28) | 5 (33) | 15 (26) | 3 (43) |  |
| Focal to bilateral tonic-clonic^5^ | 21 (72) | 9 (60) | 41 (71) | 4 (57) |  |
| Unknown | 0 (0) | 1 (7) | 2 (3) | 0 (0) |  |

ASM=Antiseizure medication
